# Electroacupuncture reduces cold stress-induced pain through microglial inactivation and transient receptor potential V1 in mice

**DOI:** 10.1186/s13020-021-00451-0

**Published:** 2021-06-03

**Authors:** Hsien-Yin Liao, Yi-Wen Lin

**Affiliations:** 1 College of Chinese Medicine, School of Post-Baccalaureate Chinese Medicine, China Medical University, Taichung, 40402 Taiwan; 2 College of Chinese Medicine, Graduate Institute of Acupuncture Science, China Medical University, 91 Hsueh-Shih Road, Taichung, 40402 Taiwan; 3 Chinese Medicine Research Center, China Medical University, Taichung, 40402 Taiwan

**Keywords:** Electroacupuncture, Fibromyalgia, Microglia, TRPV1, Hypothalamus, Cerebellum

## Abstract

**Background:**

The treatment, and efficacy thereof, is considered to be inadequate with specificity to alleviation of Fibromyalgia and its associated pain. Fibromyalgia patients suffer from chronic and persistent widespread pain and generalized tenderness. Transient receptor potential V1 (TRPV1), which is reported as a Ca^2+^ permeable ion channel that can be activated by inflammation, is reported to be involved in the development of fibromyalgia pain.

**Methods:**

The current study explored the TRPV1 channel functions as a noxious sensory input in mice cold stress model. It remains unknown whether electroacupuncture (EA) attenuates fibromyalgia pain or affects the TRPV1 pathway.

**Results:**

We show that cold stress increases mechanical and thermal pain (day 7: mechanical: 1.69 ± 0.41 g; thermal: 4.68 ± 0.56 s), and that EA and *Trpv1* deletion counter this increase. EA and *Trpv1* deletion reduced the cold stress-induced increase in inflammatory mediators and TRPV1-related molecules in the hypothalamus, periaqueductal gray (PAG), and cerebellum of mice.

**Conclusions:**

Our results imply that EA has an analgesic effect associated with TRPV1 downregulation. We provide novel evidence that these inflammatory mediators can modulate the TRPV1 signaling pathway and suggest new potential therapeutic targets for fibromyalgia pain.

## Introduction

Fibromyalgia pain lacks objective parameters for diagnosis and therapeutic effect evaluation. Its major symptoms are persistent widespread mechanical and thermal hyperalgesia and generalized tenderness. Fibromyalgia affects a large majority of the global population and is diagnosed more often in females than in males [1]. Because of a lack of etiological and pathogenic understanding of the disease development, conventional treatments are inefficient against fibromyalgia. Recent evidence implied that the central nervous system (CNS) plays an important role in the amplification of pain signals and the neurotransmitters associated with it [2]. Well-established fibromyalgia animal models can be produced by acidic saline injection into the gastrocnemius muscle [3], sound stress [4], and cold stress [5].

The high mobility group box-1 (HMGB1) is a crucial inflammatory mediator in several pain conditions and consequently often presents with an enhanced immune response observed in various pathological conditions [6, 7]. Attenuation of HMGB1 significantly reduced neuropathic pain behaviours in rats with chronic constriction injury [8, 9]. S100B (a protein released by microglia) has also been established to be involved in the inflammatory process within the CNS of rats [10]. Moreover, higher serum S100B levels correlate with a lower pressure-pain threshold in fibromyalgia patients [11]. S100B can activate the receptor for advanced glycation end-products (RAGE), which increase the Interleukin-1β (IL-1β) and Tumor Necrosis Factor-α (TNF-α) levels, thus activating the Nuclear Factor kappa-light-chain-enhancer of activated B cells (NFkB) in microglia [12]. Increased S100B in either the central or peripheral nervous system participates in inflammation [13]. In several acute and chronic diseases, S100B activates RAGE, which stimulates cox-2 expression [14].

Transient receptor potential vanilloid 1 (TRPV1) is a calcium-permeable ion channel that plays a crucial role in pain [15, 16]. TRPV1 is highly expressed in the peripheral dorsal root ganglion (DRG), spinal cord, and brain. Mechanical and thermal stimuli, acidic conditions, and capsaicin can activate TRPV1 [17]. After inflammatory pain, TRPV1 in the DRG and spinal cord levels remain elevated for 28 days [18]. Mechanical and thermal hyperalgesia was abolished in TRPV1^−/−^ mice [13, 16]. Selective antagonists of TRPV1 can significantly reduce mechanical or thermal pain sensation [19, 20]. Recently, we suggested that TRPV1 and related molecules were involved in the mice medial prefrontal cortex, hippocampus, and periaqueductal gray (PAG) following a cold stress challenge [5]. TRPV1 activation increases the protein kinases and mitogen-activated protein kinases (MAPK) that are crucial in several pain pathways. The MAPK family includes the extracellular signal-regulated protein kinase (ERK), p38, and c-Jun N-terminal kinase/stress-activated protein kinase (JNK) [21]. The TRPV1-related PI3K–Akt–mTOR axis also modulates several pain processes [22]. Toll-like receptor 4 (TLR4) is an inflammatory receptor involved in the innate and acquired immune responses. TLR4, linked with the myeloid differentiation primary response protein 88 (MyD88), can further activate NFκB for nuclear transcription. Microglial HMGB1 can bind to TLR4 and then trigger the production of IL-1β and TNF-α through the NFκB pathway, which initiates an inflammatory response [23–25].

Acupuncture is a more than 3000 years old practice that consists inserting steel needles through the skin at specific points (acupoint). Recent reports suggest that electroacupuncture (EA) can treat inflammatory pain, neuropathic pain, and fibromyalgia pain in mice [3, 5, 13, 16]. EA appears to relieve pain by increasing the release of endogenous opiates [26], dopamine [27], and adenosine [28]. EA can also reduce cold stress pain (CSP) through downregulation of interleukins, TNFα, and IFN-γ in mice plasma [5]. Our previous article suggested that EA can reduce mechanical and thermal hyperalgesia in an inflammatory mouse model by attenuating the brain TRPV1 signaling pathway [15].

In the current study, we aimed to investigate the role of TRPV1 and its related molecules in murine CSP model. The actual therapeutic effect and detailed mechanisms of EA in this CSP model remain unknown. We hypothesized that cold stress-induced inflammation activates TRPV1 and related molecules. We suggest that EA can relieve CSP by reducing inflammatory mediators, and show that EA affects the TRPV1 pathway. This study provides novel evidence on the relationship between CSP and TRPV1. We provide new evidence to support the clinical use of EA for treating fibromyalgia.

## Methods and materials

### Animals

There are totally 40 female C57BL/6 mice, aged 8–12 weeks, were used in this study. After arriving, the mice were kept in a 12 h light-dark cycle with food and water ad libitum. A sample size of ten animals per group was calculated as the number required for an alpha of 0.05 and a power of 80%. In addition, the number of animals used here and their suffering were minimized. The laboratory workers were blind to treatment allocation during the experiments and analysis. The use of these animals was approved by the Institute of Animal Care and Use Committee of China Medical University (Permit no. CMUIACUC-2019-106), Taiwan, following the Guide for the use of Laboratory Animals (National Academy Press). Mice were subdivided into four groups: Normal group (Group 1: Normal); Cold stress pain group (Group 2: CSP); 2 Hz Electroacupuncture group (Group 3: 2 Hz EA), and *Trpv1* knockout group (Group 4: *Trpv1*^−/−^). *Trpv*1^−/−^ mice were purchased (Jackson Lab, Bar Harbor, ME) and backcrossed with C57BL/6 mice for more than 10 generations.

### CSP model and Bio-Plex ELISA

All mice were host at room temperature, 24 ± 1 °C, before experiments. In the intermittent cold stress pain (CSP) model, not in normal group, 2 mice were caged in a plexiglass cage (13 × 18.8 × 29.5 cm) covered with a stainless steel mesh. On the first day (day 0), the mice were kept in a cold room at 4 °C overnight (from 4:00 pm to 10 am). The mice were next moved to 24 °C for 30 min at 10 am. After 30 min, mice were then moved back to the cold room at 4 °C for 30 min. This process was repeated for till 4:00 pm. The mice were then placed in the 4 °C cold room overnight. Normal mice were kept at room temperature from day 0 to 7 of the experiment, with no interventions applied. Mice plasma was collected and analyzed on Bio-Plex cytokine assays (BIO-RAD, CA, USA).

### EA treatments

The mice were anaesthetized with 5% isoflurane for induction, and then maintained in 1% isoflurane. Under anesthesia, a pair of stainless steel acupuncture needles (1″ in., 36G, YU KUANG, Taiwan) were bilaterally inserted at a depth of 3–4 mm into the murine equivalent of the human ZuSanLi (ST36) acupoints. The murine ST36 is located on the first dorsal interossei, radial to the midpoint of the second metacarpal bone in the forelimb. In the EA group, electrical stimuli were delivered by Trio 300 stimulator (Ito, Japan) at an intensity of 1 mA for 20 min at 2 Hz with a pulse width of 100 µs. The EA treatment caused slight visible muscle twitching around the area of insertion. The EA stimulation was applied thrice from day 5 to 7, following the CSP protocol.

### Pain behavior test

The mechanical and thermal pain behaviors were determined 3 times from day 5 to 7 throughout the experiment after the induction of the CSP model. All mice were moved to the behavior analysis room, and were adapted to the environment for at least 30 min before behavior tests. All experiments were performed at room temperature and the stimuli were applied only when the animals were calm but not sleeping or grooming. First, the von Frey filament test was conducted. Mechanical sensitivity was measured by testing the force of responses to stimulation with three applications of the electronic, calibrated von Frey filament (IITC Life Science Inc., USA). Mice were placed onto a metal mesh (75 × 25 × 45 cm) and covered with a plexiglass cage (10 × 6 × 11 cm). Subjects were then mechanically stimulated by the tip of the filament at the plantar region of the right hind paw. The filament gram counts were recorded when the stimulation caused the subject to withdraw its hind paw. Second, the Hargreaves’ assessment was used to measure thermal pain behavior by testing the time of response to thermal stimulation with three applications using Hargreaves’ test IITC analgesiometer (IITC Life Sciences, SERIES8, Model 390G). The mice were placed in a plexiglass cage on top of a glass sheet. The thermal stimulator was positioned under the glass sheet and the focus of the projection bulb was aimed exactly at the middle of the plantar surface of the right hind paw. A cut-off time of 20 s was set to prevent tissue damage. In the thermal paw withdrawal test, the nociception threshold was assessed using the latency of paw withdrawal upon stimulus, and was recorded when the constant applied heat stimulation caused the subject to withdraw its hindpaw.

### Western blot analysis

The mice were anaesthetized with 1% isoflurane and cervical dislocation. The hypothalamus, PAG, and cerebellum VI and VII tissues were immediately excised to extract proteins. Tissues were initially placed on ice and later stored at – 80 °C, pending protein extraction. Total proteins were homogenized in cold radioimmunoprecipitation (RIPA) lysis buffer containing 50 mM Tris-HCl pH 7.4, 250 mM NaCl, 1 % NP-40, 5 mM EDTA, 50 mM NaF, 1 mM Na_3_VO_4_, 0.02% NaN3, and 1× protease inhibitor cocktail (AMRESCO). The extracted proteins were subjected to 8% SDS-Tris glycine gel electrophoresis and transferred to a PVDF membrane. The membrane was blocked with 5% non-fat milk in TBS-T buffer (10 mM Tris pH 7.5, 100 mM NaCl, 0.1% Tween 20), incubated with a primary antibody in TBS-T with 1% bovine serum albumin (BSA) for 1 h at room temperature antibody against TRPV1 (∼ 95 kDa, 1:1000, Alomone, Israel), HMGB1 (∼ 28 kDa, 1:1000, Alomone, Israel), S100B (∼ 10 kDa, 1:1000, Millipore, USA), TLR4 (∼ 35 kDa, 1:1000, Millipore, USA), RAGE (∼ 42 kDa, 1:1000, Millipore, USA), pPI3K (∼ 125 kDa, 1:1000, Millipore, USA), pERK1/2 (∼ 42–44 kDa, 1:1000, Millipore, USA), pp38 (∼ 41 kDa, 1:1000, Millipore, USA), pJNK (∼ 42 kDa, 1:1000, Millipore, USA), pAkt (∼ 60 kDa, 1:1000, Millipore, USA), pmTOR (∼ 60 kDa, 1:500, Millipore, USA), and pNFB (∼ 65 kDa, 1:1000, Millipore, USA), in TBS-T with 1% bovine serum albumin. Peroxidase-conjugated anti-rabbit antibody, anti-mouse antibody or anti-goat antibody (1: 5000) was used as the appropriate secondary antibody. The bands were visualized by an enhanced chemiluminescent substrate kit (PIERCE) with LAS-3000 Fujifilm (Fuji Photo Film Co., Ltd.). Where applicable, the image intensities of specific bands were quantified with NIH ImageJ software (Bethesda, MD, USA). β-actin or α-tubulin was utilized as internal control.

### Immunofluorescence

Mice were euthanized with a 5% isoflurane via inhalation and intracardially perfused with normal saline followed by 4% paraformaldehyde. The brain was immediately dissected and post fixed with 4% paraformaldehyde at 4 ºC for 3 days. The tissues were placed in 30% sucrose for cryoprotection overnight at 4 ºC. The brain was embedded in an Optimal cutting temperature (OCT) compound and rapidly frozen using liquid nitrogen before storing the tissues at – 80 ºC. Frozen segments were cut at 20-µm width on a cryostat then instantaneously placed on glass slides. The samples were fixed with 4% paraformaldehyde, then incubated with a blocking solution, consisting of 3% BSA, 0.1 % Triton X-100, and 0.02% sodium azide, for 1 h at room temperature. After blocking, the samples were incubated with the primary antibody (1:200, Alomone), TRPV1 and Iba1, prepared in 1% bovine serum albumin solution at 4 ºC overnight. The samples were then incubated with the secondary antibody (1:500), 488-conjugated AffiniPure donkey anti-rabbit IgG (H+L), 594-conjugated AffiniPure donkey anti-goat IgG (H+L) and Peroxidase-conjugated AffiniPure donkey anti-mouse IgG (H+L) for 2 h at room temperature before being fixed with cover slips for immunofluorescence visualization. The samples were observed by an epi-fluorescent microscope (Olympus, BX-51, Japan) with 20× numerical aperture (NA = 1.4) objective. The images were analyzed by NIH ImageJ software (Bethesda, MD, USA).

### Statistical analysis

Statistical analysis was performed using the SPSS statistic program. All statistic data are presented as the mean ± standard error (SEM). Shapiro–Wilk test was performed to test the normality of data. Statistical significance among all groups was tested using the repeated measure ANOVA test, followed by a post hoc Tukey’s test. Values of p < 0.05 were considered statistically significant.

## Results

### Electroacupuncture inhibits cold stress-induced pain in mice

**Fig. 1:**
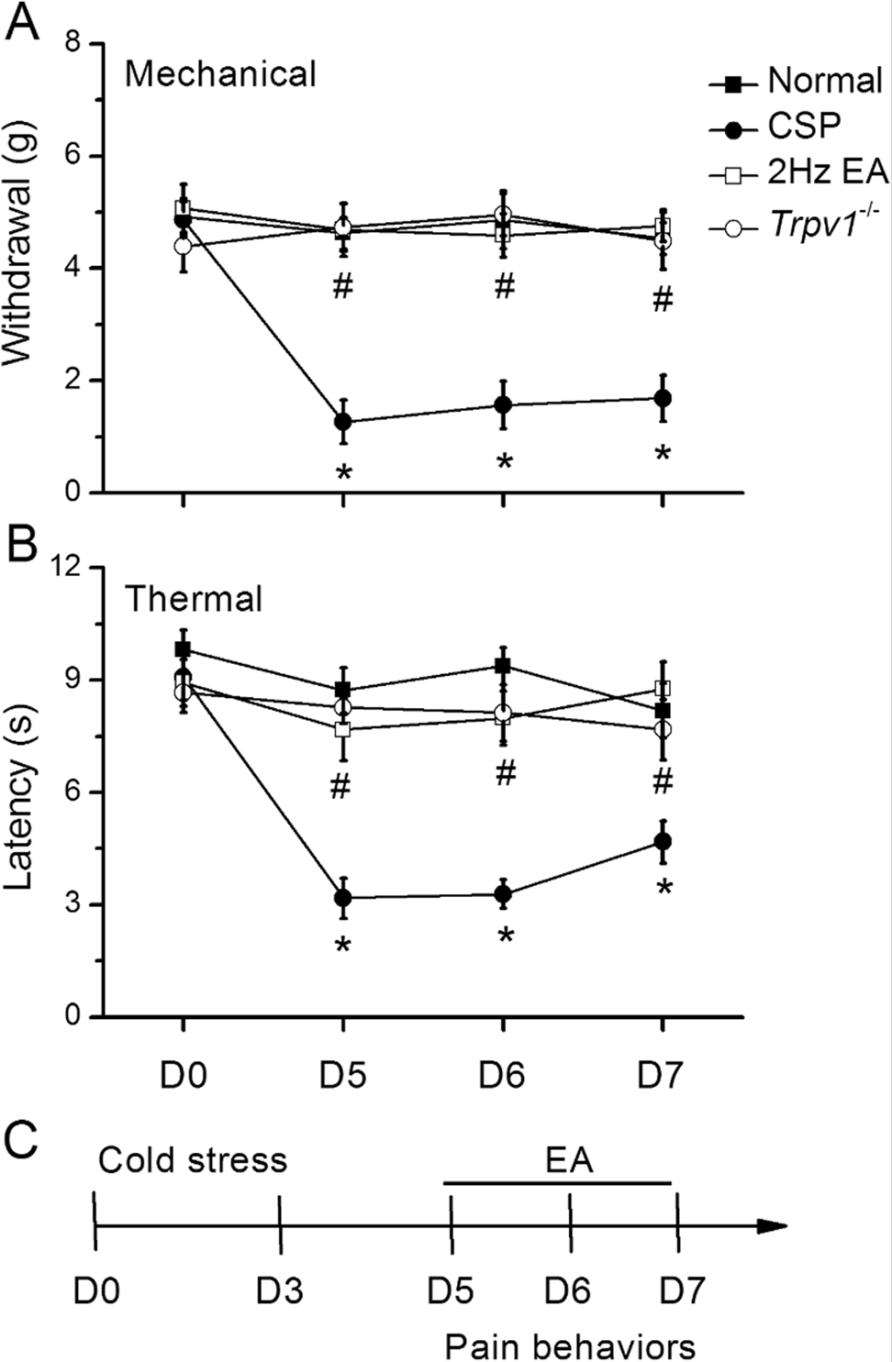
Mechanical withdrawal, thermal latency, and experimental flow in normal, CSP, EA, and *Trpv1*^−/−^ mice. **A** Mechanical threshold from the von Frey tests. **B** Thermal latency from the Hargreaves’ test. **C** Experimental flow in normal, CSP, EA, and *Trpv1*^−/−^ mice. *Indicates statistical significance when compared with the normal group. ^#^Indicates statistical significance when compared with the CSP groups. n = 10 in all groups

To test the efficacy of EA in alleviating CSP induced mechanical and thermal hyperalgesia, we compared responses of the von Frey filament and Hargraves’ test among all the groups. Before CSP induction, all mice had similar mechanical responses that showed normal distribution and no statistical significance between each group. Mechanical hyperalgesia was observed in the CSP mice (Fig. 1A, *p < 0.05, black circles, 1.69 ± 0.41, n = 10). This result indicated a successful induction of CSP pain. The von Frey test revealed that EA and *Trpv1* deletion substantially attenuated the typical intermediate cold stress-induced mechanical hyperalgesia (Fig. 1A, D7: EA group: 4.76 ± 0.28, *Trpv1*^−/−^ group: 4.49 ± 0.51, n = 10). Next, we examined whether EA or *Trpv1* deletion also altered thermal hyperalgesia in CSP mice. The Hargraves’ test revealed significant thermal hyperalgesia (paw withdrawal latency) after cold stress induction (Fig. 1B, D2: 4.68 ± 0.56, n = 10), accordingly providing further evidence to indicate successful CSP pain induction. EA and *Trpv1* deletion reversed the latency decrease further (Fig. 1A, D7: EA group: 8.76 ± 0.72, *Trpv1*^*−/−*^ group: 7.68 ± 0.81). Figure 1C illustrates the experimental protocol.

**Fig. 2:**
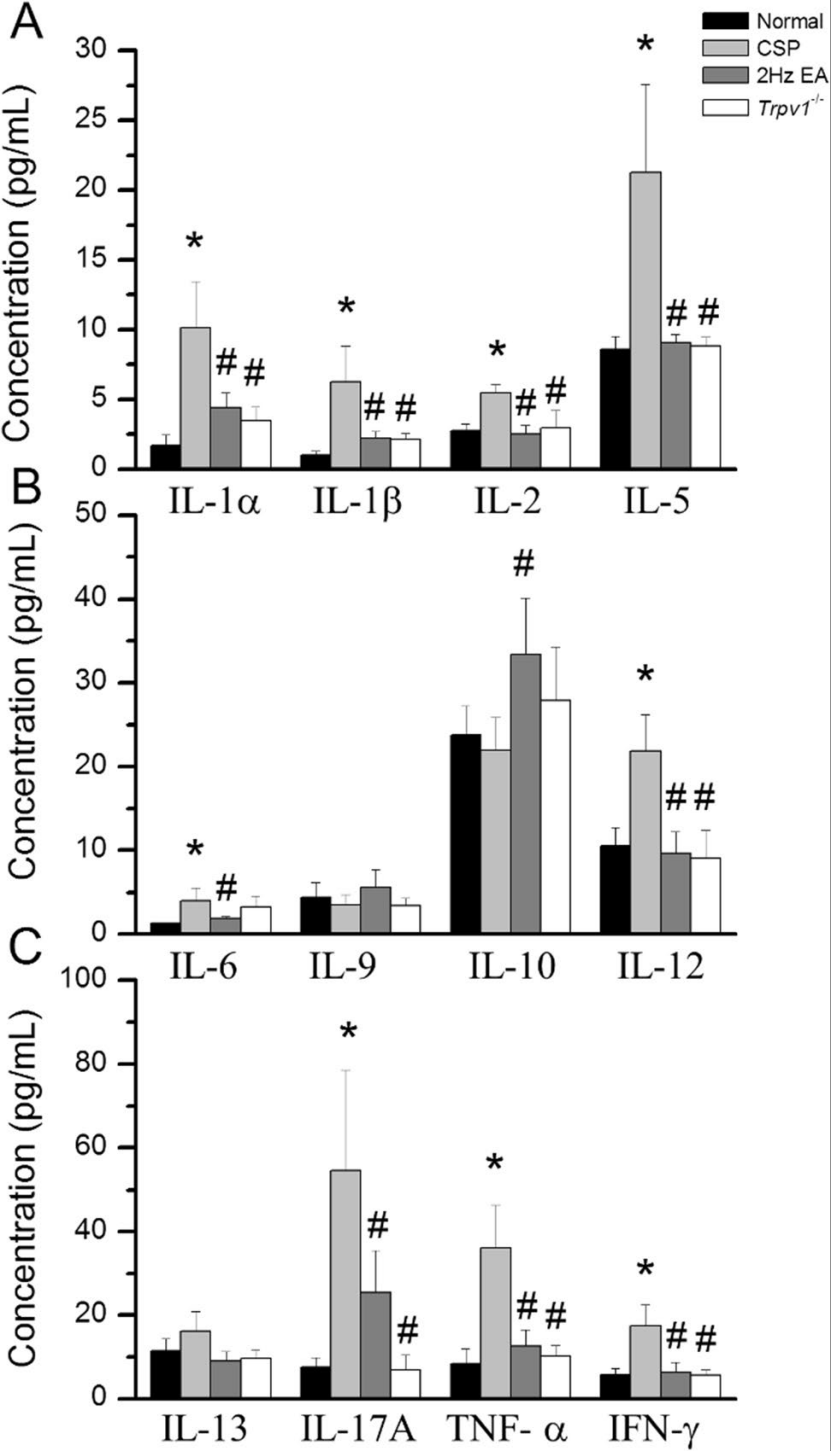
Inflammatory mediators plasma concentrations in mice. **A** IL-1α, IL-1β, IL-2, and IL-5, **B** IL-6, IL-9, IL-10, and IL-12, **C** IL-13, IL-17 A, TNF-α, and IFN-γ. **Indicates statistical significance when compared with the normal group. ^#^Indicates statistical significance when compared with the CSP group. *IL* Interleukin, *IFN* Interferon, *TNF* Tumor necrosis factor. n = 6 in all groups

### EA and *Trpv1* deletion reversed the cold stress-induced increase in inflammatory mediators

**Fig. 3:**
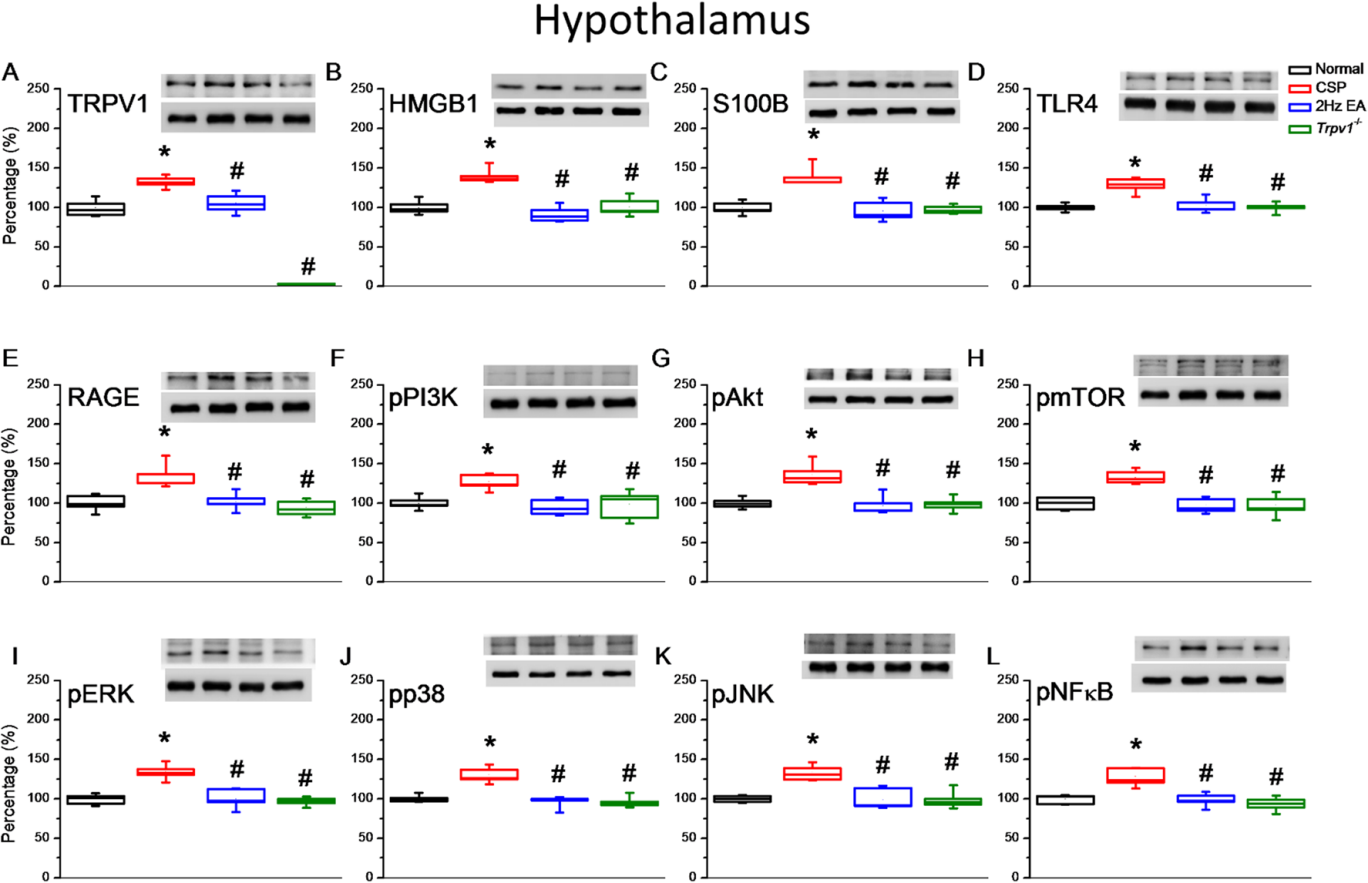
Levels of TRPV1 and related molecules in the mice hypothalamus. The western blot bands contain four lanes of protein expression corresponding to the Normal, CSP, EA, and *Trpv1*^−/−^ groups. **A** TRPV1, **B** HMGB1, **C** S100B, **D** TLR4, **E** RAGE, **F** pPI3K, **G** pAkt, **H** pmTOR, **I** pERK, **J** pp38, **K** pJNK, and **L** pNFκB protein levels. *Indicates statistical significance when compared with the normal group. ^#^Indicates statistical significance when compared with the CSP group. n = 6 in all groups

To test the role of inflammatory mediators in CSP mice, we quantified them in mouse plasma using a Bio-Plex ELISA technique. CSP mice had higher levels of inflammatory mediators IL-1α, IL-1β, IL-2, IL-5, IL-6, IL-12, IL-17 A, TNF-α, and IFN-γ than normal mice (Fig. 3, *p < 0.05, n = 6, light gray column). EA and *Trpv1* deletion dramatically lowered these levels (Fig. 3, EA group: ^#^*p* < 0.05, n = 6, gray column, *Trpv1*^*−/−*^ group: ^#^*p* < 0.05, n = 6, white column).

### EA or *Trpv1* deletion reduced CSP through TRPV1 signaling pathways in the mice hypothalamus

**Fig. 4:**
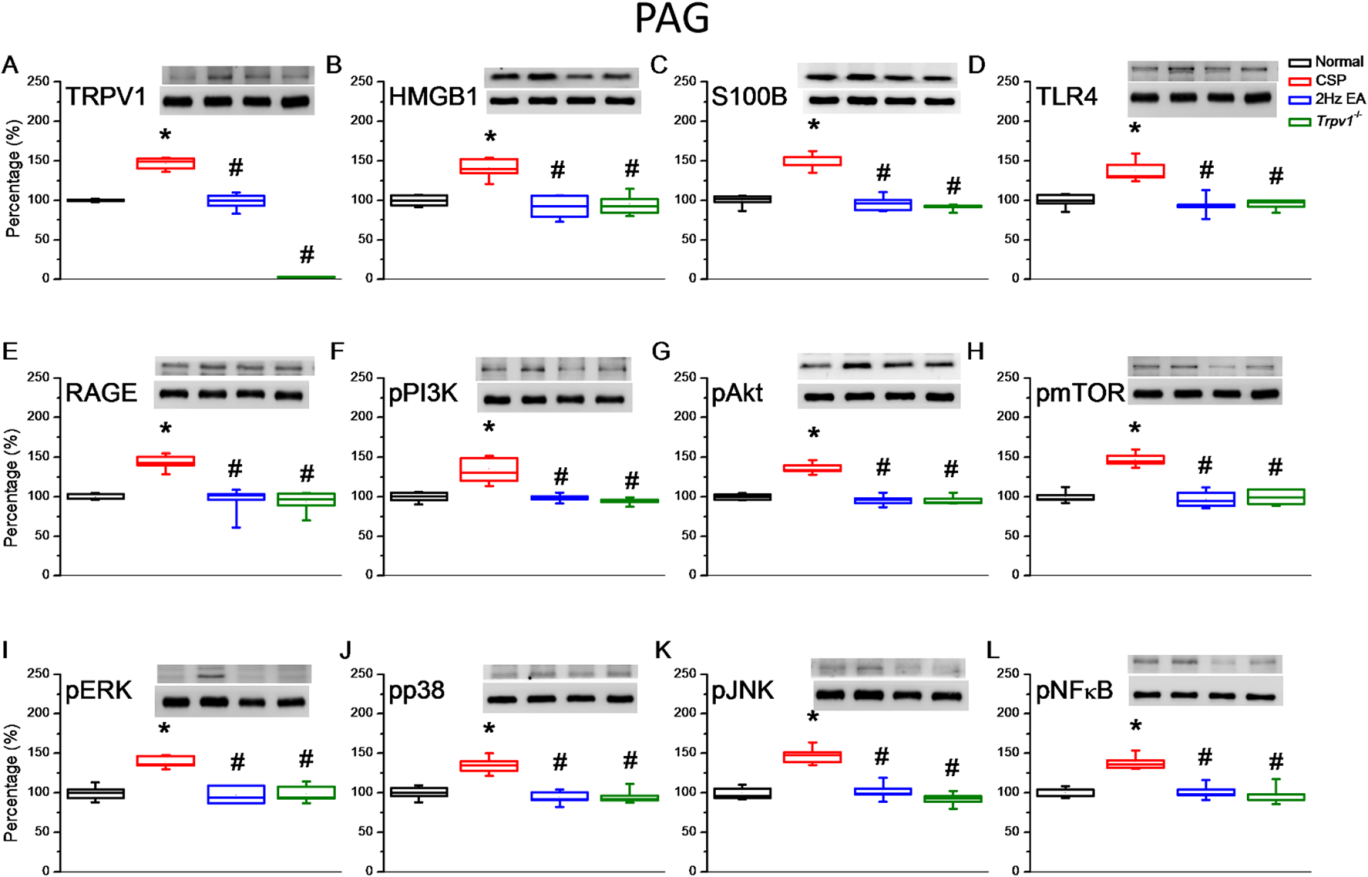
Levels of TRPV1 and related molecules in the mice PAG. The western blot bands contain four lanes of protein expression corresponding to the Normal, CSP, EA, and *Trpv1*^−/−^ groups. **A** TRPV1, **B** HMGB1, **C** S100B, **D** TLR4, **E** RAGE, **F** pPI3K, **G** pAkt, **H** pmTOR, **I** pERK, **J** pp38, **K** pJNK, and **L** pNFκB protein levels. *Indicates statistical significance when compared with the normal group. ^#^Indicates statistical significance when compared with the CSP group. n = 6 in all groups

Using Western blot, we quantified actors of the TRPV1 signaling pathway in the mouse hypothalamus. CSP mice had significantly higher levels of TRPV1 than normal mice (Fig. 4A, red column, *p < 0.05, n = 6). EA significantly reduced TRPV1 levels (Fig. 4A, blue column, ^#^*p* < 0.05, n = 6). As expected, *Trpv1*^−/−^ mice did not express TRPV1 (Fig. 4A, green column, ^#^*p* < 0.05, n = 6). We next measured the expression levels of the inflammation mediators HMGB1 and S100B. Similarly to TRPV1, the levels of HMGB1 and S100B were expectantly higher in the CSP group (Fig. 4B, C, *p < 0.05, n = 6) than in the EA and *Trpv1*^−/−^ groups (Fig. 4B, C, ^#^*p* < 0.05, n = 6). Besides, we measured the expression levels of TLR4 and RAGE, which are receptors for HMGB1 and S100B, respectively. The CSP group had higher hypothalamus levels of TLR4 and RAGE (Fig. 4D, E, *p < 0.05, n = 6) than the EA and *Trpv1*^*−/−*^ groups (Fig. 4D, E, ^#^*p* < 0.05, n = 6). Furthermore, the CSP group had higher hypothalamus levels of downstream molecules such as pPI3K, pAkt, and pmTOR (Fig. 4F–H, *p < 0.05, n = 6) than the EA and *Trpv1*^−/−^ groups (Fig. 4F–H, ^#^*p* < 0.05, n = 6). Next, we measured the expression of pERK, pp38, and pJNK to check whether the MAPK family was involved in this model. The CSP group had higher levels of pERK, pp38, and pJNK than the normal group (Fig. 4I–K, *p < 0.05, n = 6). The EA and *Trpv1*^−/−^ groups had significantly lower levels than the CSP group (Fig. 4I–K, ^#^*p* < 0.05, n = 6). Finally, we measured the levels of the transcriptional factor pNFκB in the hypothalamus. The CSP group had higher pNFκB levels than the normal group (Fig. 4L, *p < 0.05, n = 6), EA, and *Trpv1*^−/−^ groups (Fig. 4L, ^#^*p* < 0.05, n = 6). These results support the participation of the inflammatory and TRPV1 pathways in the CSP model. Moreover, EA and *Trpv1* deletion reversed the CSP-induced overexpression of TRPV1 and related molecules.

### EA and *Trpv1* deletion reversed the cold stress-induced increase in inflammatory mediators and TRPV1 signaling pathway in the PAG

**Fig. 5:**
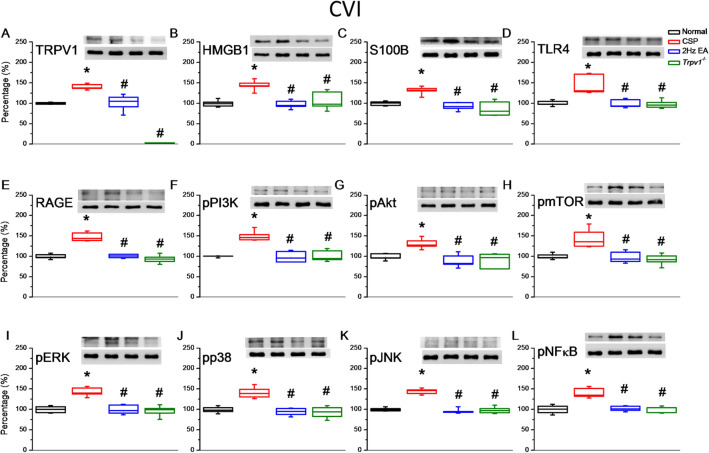
Levels of TRPV1 and related molecules in the mice cerebellum CVI. The western blot bands contain four lanes of protein expression corresponding to the Normal, CSP, EA, and *Trpv1*^−/−^ groups. **A** TRPV1, **B** HMGB1, **C** S100B, **D** TLR4, **E** RAGE, **F** pPI3K, **G** pAkt, **H** pmTOR, **I** pERK, **J** pp38, **K** pJNK, and **L** pNFκB protein levels. *Indicates statistical significance when compared with the normal group. ^#^Indicates statistical significance when compared with the CSP group. n = 6 in all groups

Since PAG plays crucial roles in pain processing, we checked whether cold stress affected the inflammatory mediators and TRPV1 signaling pathway in the PAG using Western blot. Cold stress notably increased TRPV1 expression in the PAG (Fig. 5A, red column, *p < 0.05, n = 6). EA significantly reduced this increase (Fig. 5A, blue column, ^#^p < 0.05, n = 6). As expected, *Trpv1*^−/−^ mice did not express TRPV1 (Fig. 5A, green column, ^#^p < 0.05, n = 6). Cold stress also increased HMGB1 and S100B levels (Fig. 5B, C, red column, *p < 0.05, n = 6). However, the EA and *Trpv1*^−/−^ groups had significantly lower HMGB1 and S100B levels (Fig. 5B, C, blue and green column, ^#^p < 0.05, n = 6). To further evaluate the roles of TLR4 and RAGE in CSP modulation, we observed that EA and *Trpv1* deletion reversed CSP-induced overexpression (Fig. 5D, E, blue and green column, ^#^p < 0.05, n = 6). Similarly, EA and *Trpv1* deletion reversed the increase of the pPI3K–pAkt–pmTOR axis downstream molecules observed in the CSP group (Fig. 5F–H, p < 0.05, n = 6). Cold stress also increased the MAPKs, pERK, pp38, and pJNK levels, and EA and *Trpv1* deletion reversed this increase. (Fig. 5I–K, p < 0.05, n = 6). We observed a similar pattern for the transcriptional factor pNFκB (Fig. 5L, p < 0.05, n = 6).

### The effect of EA and *Trpv1* deletion on nociceptor and its downstream molecules in the cerebellar lobules VI and VII

**Fig. 6:**
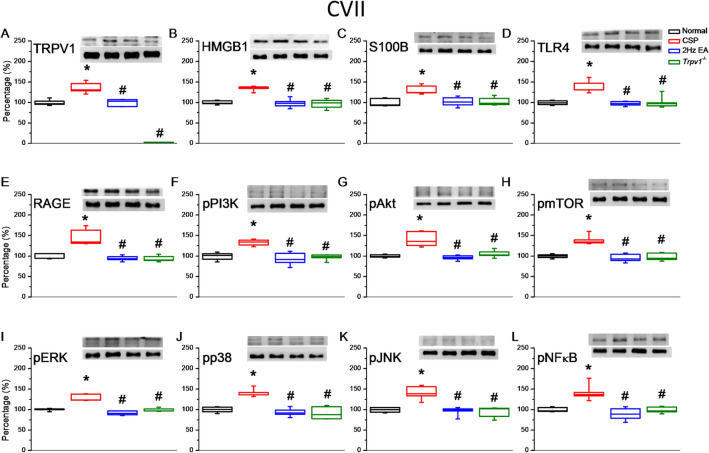
Levels of TRPV1 and related molecules in the mice cerebellum CVII. The western blot bands contain four lanes of protein expression corresponding to the Normal, CSP, EA, and *Trpv1*^−/−^ groups. **A** TRPV1, **B** HMGB1, **C** S100B, **D** TLR4, **E** RAGE, **F** pPI3K, **G** pAkt, **H** pmTOR, **I** pERK, **J** pp38, **K** pJNK, and **L** pNFκB protein levels. *Indicates statistical significance when compared with the normal group. ^#^Indicates statistical significance when compared with the CSP group. n = 6 in all groups

**Fig. 7:**
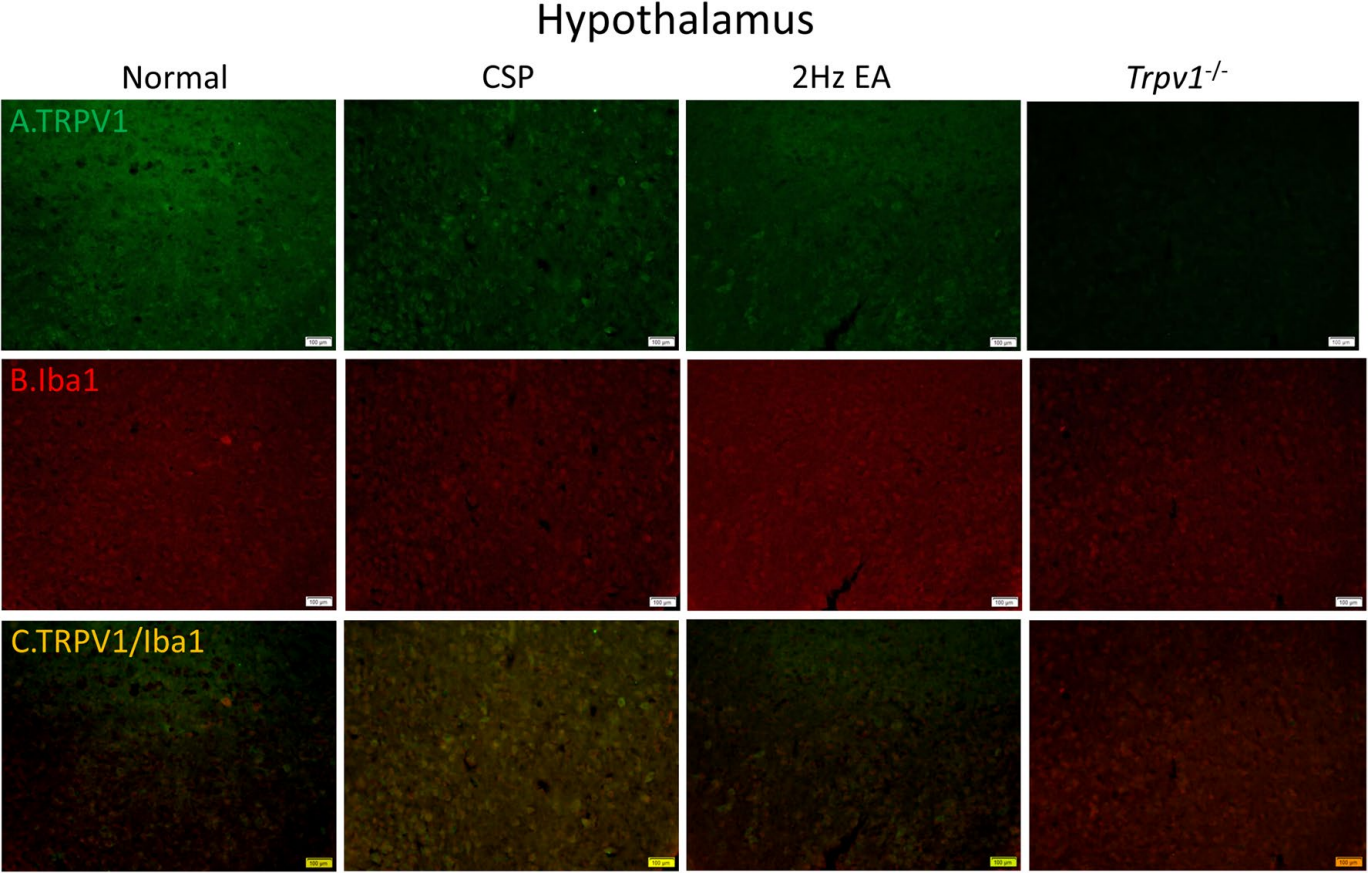
Immunofluorescence staining of TRPV1, Iba1, and double staining protein expression in the mice hypothalamus. **A** TRPV1, **B** Iba1, and **C** TRPV1/Iba1 double staining, immuno-positive (green, red, or yellow) signals in the mice hypothalamus region. Scale bar: 100 μm. n = 4 in all groups

After CSP induction, we collected cerebellum samples to measure protein levels in the cerebellar lobules VI and VII. Cold stress significantly increased the levels of TRPV1, HMGB1, S100B, TLR4, and RAGE. EA and *Trpv1* deletion reversed the overexpression (Fig. 6, n = 6). We observed a similar pattern for pPI3K, pAkt, and pmTOR. The CSP group also had higher levels of pERK, pp38, and pJNK than the normal group. Again, EA and *Trpv1* deletion reversed these increases. Finally, we observed a similar pattern for pNFκB, which functions inside the nucleus. Similar results were also observed in the cerebellar lobule VII (Fig. 7, n = 6).

### Effect of EA *Trpv1* deletion on protein expression in the hypothalamus and PAG

**Fig. 8:**
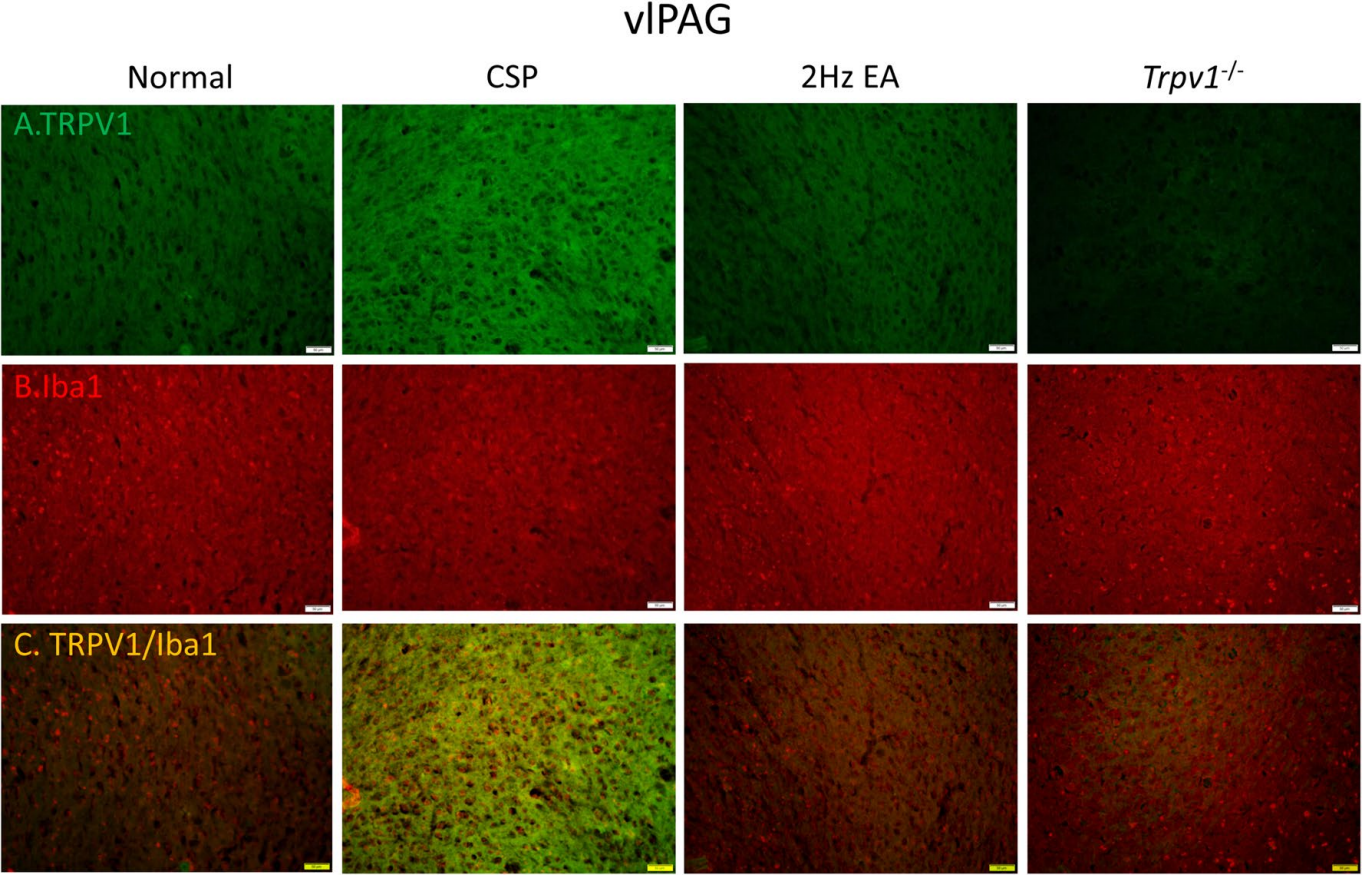
Immunofluorescence staining of TRPV1, Iba1, and double staining protein expression in the mice ventrolateral PAG. **A** TRPV1, **B** Iba1, and **C** TRPV1/Iba1 double staining, immuno-positive (green, red, or yellow) signals in the mice vlPAG region. Scale bar: 100 μm. n = 4 in all groups

To determine neuronal or microglial mechanisms by which TRPV1 modulates CSP, we stained TRPV1 and Iba1 protein expression in the mouse hypothalamus and ventral lateral PAG. As shown in Fig. 2, the CSP group had both higher hypothalamus levels of TRPV1 (Fig. 2A) and Iba1 (Fig. 2B) than the normal group. EA significantly reduced the protein density. We observed no signal in the *Trpv1*^−/−^ group (Fig. 2A). Similar results were observed for TRPV1 and Iba1 expression in the mice ventral lateral PAG (Fig. 8A, B). Moreover, we observed increased double-stained immune-positive signals in the CSP group suggesting colocolization of TRPV1 and Iba1 (Figs. 2 and 8C). EA and *Trpv1* deletion attenuated these phenomena.

### EA and *Trpv1* deletion significantly attenuated the increase of TRPV1 and Iba1 in the mice cerebellar lobules VI and VII

EA and *Trpv1* deletion significantly attenuated the increase of TRPV1 and Iba1 in the mice cerebellar lobules VI and VII

**Fig. 9:**
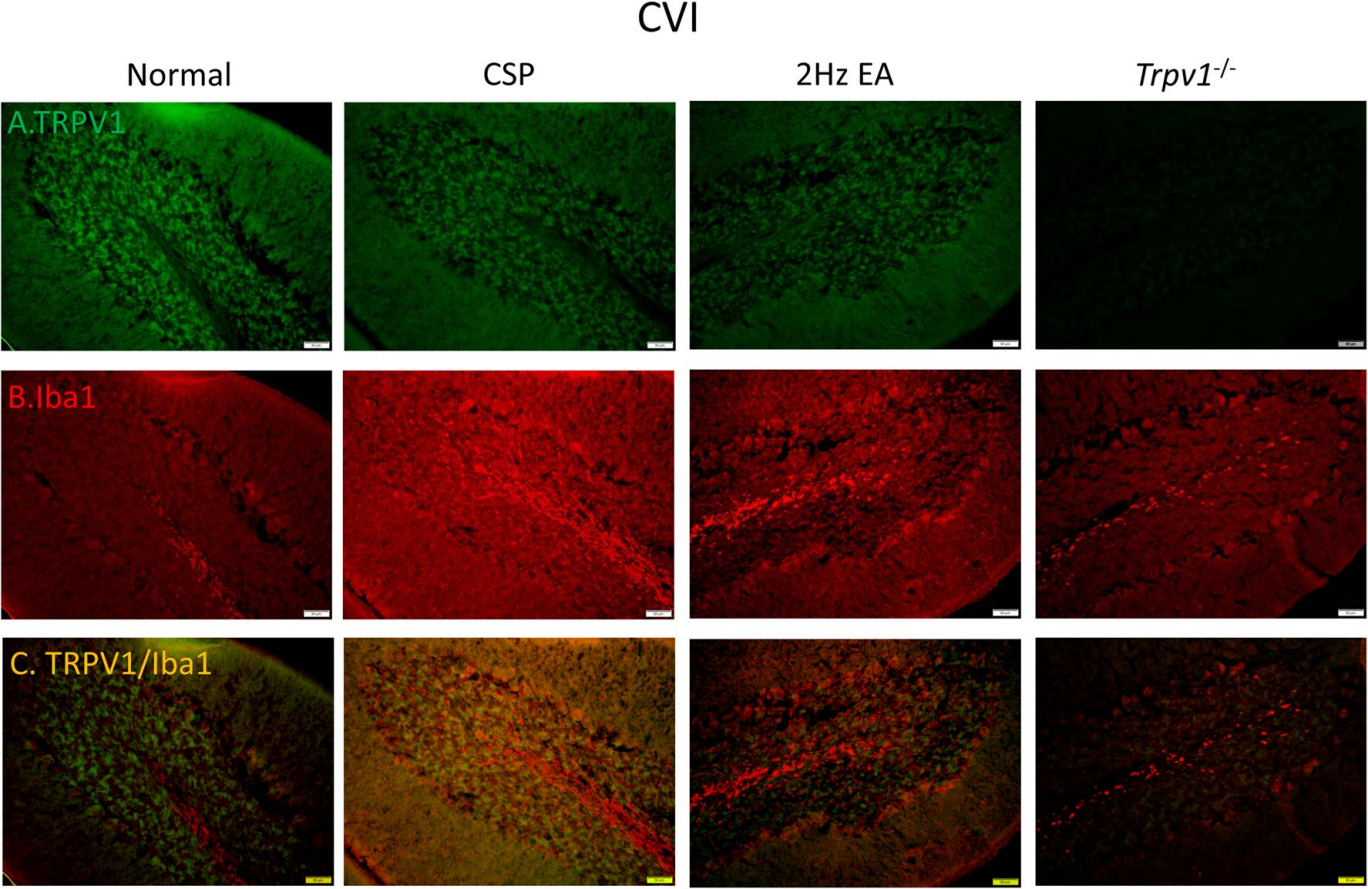
Immunofluorescence staining of TRPV1, Iba1, and double staining protein expression in the mice cerebellar lobule VI. **A** TRPV1, **B** Iba1, and **C** TRPV1/Iba1 double staining, immuno-positive (green, red, or yellow) signals in the mice cerebellar lobule VI region. Scale bar: 100 μm. n = 4 in all groups

**Fig. 10:**
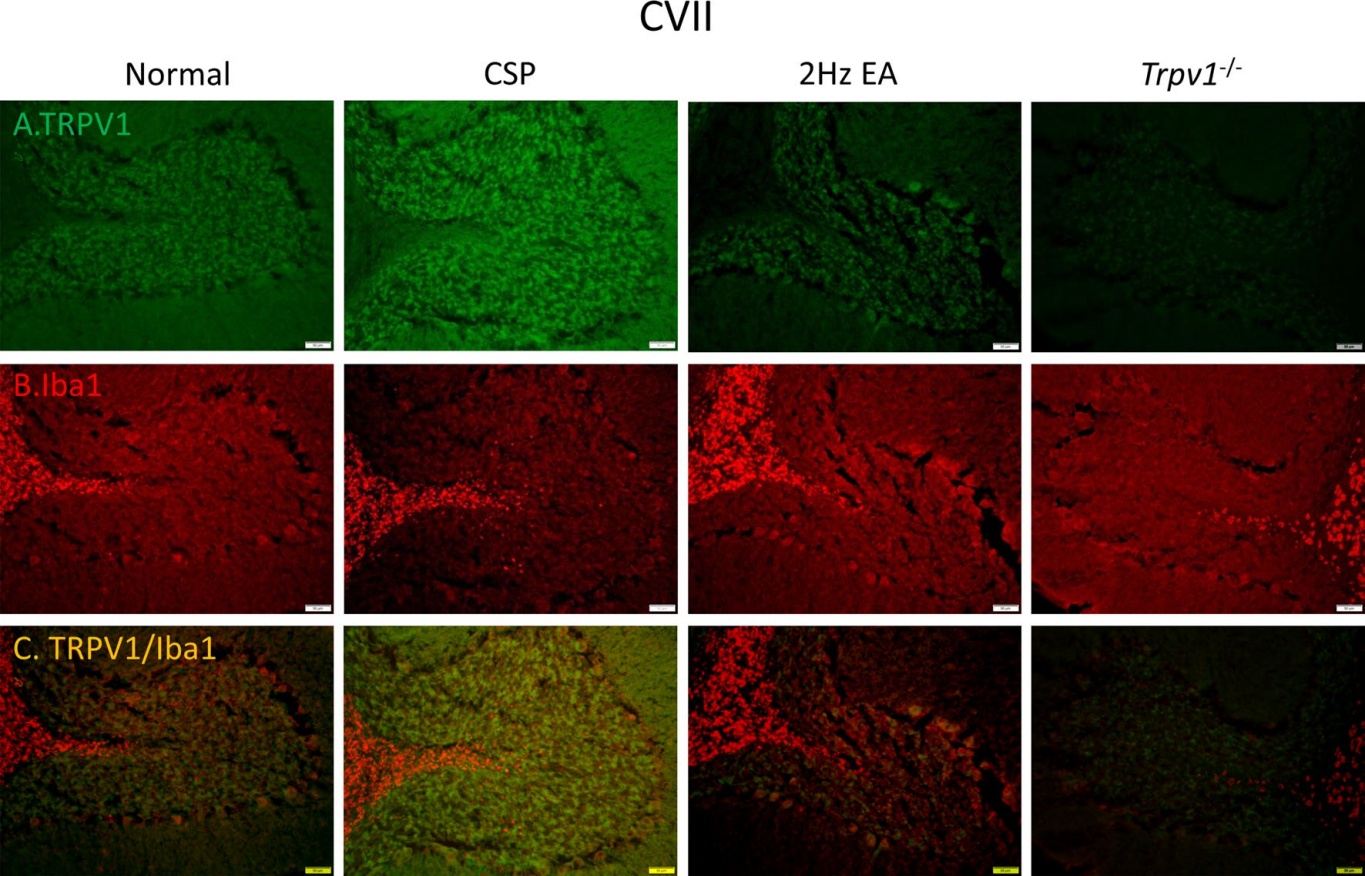
Immunofluorescence staining of TRPV1, Iba1, and double staining protein expression in the mice cerebellar lobule VII. **A** TRPV1, **B** Iba1, and **C** TRPV1/Iba1 double staining, immuno-positive (green, red, or yellow) signals in the mice cerebellar lobule VII region. Scale bar: 100 μm. n = 4 in all groups

We next focused on the cerebellum, a brain region involved in fibromyalgia pain processing, using immunostaining. We indicated that CSP would increase TRPV1 expression (Fig. 9). EA and *Trpv1* deletion visibly reduced the overexpression of TRPV1 (Fig. 9A). As Fig. 9B shows, we observed a similar pattern for Iba1. We obtained similar results for TRPV1 and Iba1 in the cerebellar lobule VII (Fig. 10A, B). Furthermore, we observed increased double-positive staining signals in the CSP group, suggesting a colocolization of TRPV1 and Iba1 (Fig. 10C). The EA and *Trpv1* deletion abrogated these signals (Fig. 10).

## Discussion

The salient finding in the current study is that cold stress activates pain, inflammation, and central sensitization pathways in mice. Cold stress caused mechanical and thermal hyperalgesia and increased IL-1α, IL-1β, IL-2, IL-5, IL-6, IL-12, IL-17A, TNF-α, and IFN-γ plasma levels. We also demonstrated that cold stress increased the expression ofTRPV1 and related molecules in the mice hypothalamus, PAG, and cerebellum. Thus, TRPV1 is an inflammatory inflammation marker of CSP. Besides, cold stress increased the release of inflammatory modulators such as HMGB1 and S100B, which participate in the pain process by activating TLR4 and RAGE. Cold stress also increased the expression of PI3K-Aky-mTOR, MAPK, and NfκB, which are downstream of TRPV1 and involved in pain signaling in the brain regions we observed. EA or *Trpv1* deletion potently suppressed these complicated molecular pathways in the CSP mice brain.

A recent study indicated that prolonged hyperalgesia is a functional pain symptom induced by intermittent cold stress and is similar to clinical fibromyalgia-like pain [22, 29]. Besides, fibromyalgia patients often have increased inflammatory mediators in peripheral circulation and cerebral spinal fluid [30]. Inflammatory mediators such as IL-1β, IL-6, and TNF-α are mainly produced by non-neuronal cells such as astrocytes and microglia. Chronic unpredictable mild stress can induce depression in mice and increase serum cytokines such as IL-1β, IL6, and TNFα [31, 32]. Recently, researchers suggested that peripheral nerve injury, CNS trauma, and nociceptive DRG neurons increased IL-1β expression [33]. Furthermore, spared nerve injury increased IL-1β expression in the plasma, spinal dorsal horn, hippocampus, prefrontal cortex, and amygdala [34]. Remarkably, neuropathic pain-inducing nerve injury increased IL-6 expression [31, 32]. Injection of IL-6 antibody reliably attenuated peripheral nerve injury-induced mechanical hyperalgesia [35]. Increased inflammation reliably increases the secretion of HMGB1 and S100B, which activate TLR4 and RAGE, participating in pain signaling [13, 16]. In this study, we used a bio-plex technique to analyze many inflammatory mediators in mice plasma. We showed that cold stress increased IL-1α, IL-1β, IL-2, IL-5, IL-6, IL-12, IL-17 A, IFN-γ, and TNF-α in mouse. EA and *Trpv1* deletion reversed these patterns.

We aimed to determine the therapeutic effect of EA using an effective fibromyalgia mouse model, which significantly increased the expressions of TRPV1 signaling pathway effectors in the hypothalamus, PAG, and cerebellum regions. EA significantly decreased the expressions of molecules related to the TRPV1 pathway, and the neuromodulatory effects of ST36 stimulation suggest that EA can have a therapeutic effect. EA acts on the psychosomatic aspects of nociceptive responses and modulates neural activity at multiple levels of the cerebellar and limbic systems [36]. Our results also showed that intermittent cold stress causes mechanical hyperalgesia through inflammatory mediators and TRPV1 pathways and that EA can improve various components of the response CSP, greatly improving the inflammatory and neuromodulatory responses. Inflammation and dysfunction of the hypothalamic–pituitary–adrenal axis have been reported in the pathogenesis of fibromyalgia [37]. pERK has a role in fibromyalgia pain and is expressed in both the peripheral and central nervous systems [38]. Besides, *Trpv1* deletion prevented the increase in pERK expression in fibromyalgia mice and displayed positive tendencies toward EA treatment in the DRG and spinal cord of fibromyalgia mice [39]. We observed the same phenomenon. EA and *Trpv1* deletion prevented the cold stress-induced increase of pERK expression in the hypothalamus, PAG, and cerebellum of mice. Besides, inflammation can increase pNFκB levels and cause hypothalamic-pituitary-adrenal axis dysfunction through cytokine involvement, which regulates the molecules and pathways associated with nociceptive signaling cascades [40, 41]. Our data also indicate an upregulation of pNFκB in the mice hypothalamus, PAG, and cerebellum, all of which are comparatively attenuated in the EA and *Trpv1*^−/−^ groups, suggesting an involvement of the TRPV1 ion channel pathway in these phenomena.

TRPV1 antagonists, *Trpv1* deletion, and EA all act similarly by decreasing pain signaling related to this channel [42, 43]. TRPV1 agonists can initiate pain but not in *Trpv1*^−/−^ mice [44, 45]. Furthermore, RNA interference *Trpv1* knockdown, and pharmacological inhibition experiments clarified the role of TRPV1 in pain pathways [46, 47]. What is the real biological significance of EA and TRPV1 in attenuating cold stress-induced inflammation and pain? Pain and inflammation are often associated. TRPV1 can shut off both inflammation and pain signals in the mice brain. Our findings suggest that EA or *Trpv1* deletion can reverse cold stress-induced pain and inflammation. Conversely, TRPV1 in the peripheral site is important for pain sensation.

## Conclusions

**Fig. 11:**
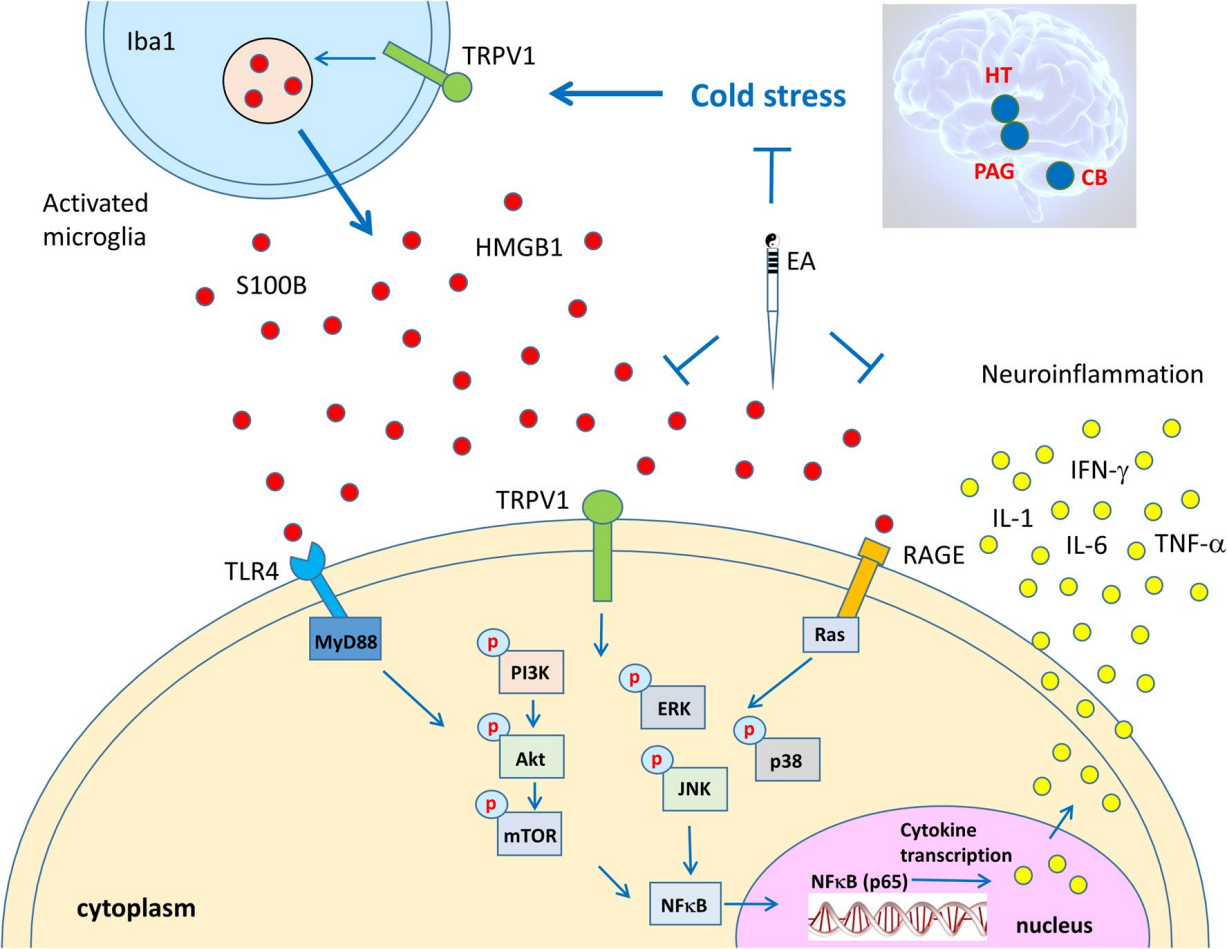
TRPV1 and related molecular pathways in the mouse brain

Our results suggest that cold stress activated pain, inflammation, and TRPV1 signaling. We observed increased expression of TRPV1 and related molecules in the mice hypothalamus, PAG, and cerebellum. EA and *Trpv1* deletion reversed this increase. This study shows that TRPV1 and related molecules play crucial roles in CSP (Fig. 11). This study will help develop future TRPV1-targeted pain treatments.

## Data Availability

The datasets supporting the conclusions of this article are included within the article.

## Abbreviations

TRPV1: Transient receptor potential V1
PAG: Periaqueductal gray
CNS: Central nervous system
HMGB1: High mobility group box-1
RAGE: Receptor for advanced glycation end-products
IL-1β: Interleukin-1β
TNF-α: Tumor necrosis factor-α
NFκB: Nuclear Factor kappa-light-chain-enhancer of activated B cells
DRG: Dorsal root ganglion
MAPK: Mitogen-activated protein kinases
ERK: Extracellular signal-regulated protein kinase
JNK: c-Jun N-terminal kinase/stress-activated protein kinase
TLR4: Toll-like receptor 4
MyD88: Myeloid differentiation primary response protein 88
EA: Electroacupuncture
CSP: Cold stress pain
